# Retinoic Acid Receptor Gamma (RAR*γ*) Promotes Cartilage Destruction through Positive Feedback Activation of NF-*κ*B Pathway in Human Osteoarthritis

**DOI:** 10.1155/2022/1875736

**Published:** 2022-11-07

**Authors:** Yue-Wei Yu, Si-Yang Li, Lin-Jun Zhang, Qian-Liang Wang, Zhong-Guo Liu, Qing-Zhi Chen, Hong-Yu Song, Dong-Yan Shen, Jun Yan

**Affiliations:** ^1^Department of Orthopedics, The Second Affiliated Hospital of Soochow University, Suzhou 215004, China; ^2^Department of Orthopedics, The First Affiliated Hospital of Xiamen University (Tongan Branch), Xiamen 361000, China; ^3^Xiamen Cell Therapy Research Center, The First Affiliated Hospital of Xiamen University, School of Medicine, Xiamen University, Xiamen 361003, China

## Abstract

Osteoarthritis (OA) is a severe inflammation-related disease which leads to cartilage destruction. The retinoic acid receptor gamma (RAR*γ*) has been indicated to be involved in many inflammation processes. However, the role and mechanism of RAR*γ* in cartilage destruction caused by inflammation in OA are still unknown. Here, we demonstrated that the RAR*γ* was highly expressed in chondrocytes of OA patients compared with healthy people and was positively correlated with the damage degree of cartilage in OA. Cytokine TNF-*α* promoted the transcription and expression of RAR*γ* through activating the NF-*κ*B pathway in OA cartilage. In addition, the overexpression of RAR*γ* resulted in the upregulation of matrix degradation and inflammation associated genes and downregulation of differentiation and collagen production genes in human normal chondrocyte C28/I2 cells. Mechanistically, overexpression of RAR*γ* could increase the level of p-I*κ*B*α* and p-P65 to regulate the expression of downstream genes. RAR*γ* and I*κ*B*α* also could interact with each other and had the same localization in C28/I2 cells. Moreover, the SD rats OA model induced by monosodium iodoacetate indicated that CD437 (RAR*γ* agonist) and TNF-*α* accelerated the OA progression, including more severe cartilage layer destruction, larger knee joint diameter, and higher serum ALP levels, while LY2955303 (RAR*γ* inhibitor) showed the opposite result. RAR*γ* was also highly expressed in OA group and even higher in TNF-*α* group. In conclusion, RAR*γ*/NF-*κ*B positive feedback loop was activated by TNF-*α* in chondrocyte to promote cartilage destruction. Our data not only propose a novel and precise molecular mechanism for OA disease but also provide a prospective strategy for the treatment.

## 1. Introduction

Osteoarthritis (OA), the most common type of arthritis, is a chronic degenerative joint disease. The main signs and symptoms of OA include pain, deformity, and dysfunction of the joints, which ultimately leaves millions of people worldwide suffering from severe pain and physical disability and even lead to serious economic and social burden [1–3]. The disease is an active dynamic change caused by an imbalance between the repair and destruction of joint tissue, rather than the passive degenerative disease and so called wear-and-tear disease that is often described [4]. OA possesses specific histopathological features such as chondrocyte loss, cartilage matrix degradation, synovial inflammation, and subchondral bone remodelling, which depend on its stage [5, 6]. The pathogenesis of OA is complex, where aging, gender, obesity, genetic susceptibility, inflammation, and certain metabolic diseases may be putative factors, however, the exact pathological mechanisms remain unclear [7]. Attributing to lack of full understanding of the pathogenesis of OA, there are currently no disease-modifying OA drugs (DMOADs) with demonstrated long-term efficacy in OA patients [8].

In the progression of OA, increased secretion of proinflammatory cytokines by abnormal synovial fibroblasts, such as IL-6, IL-1*β*, and TNF-*α*, which can activate both classical and nonclassical NF-*κ*B and IL-6/STAT3 inflammatory pathways [9]. They resulted in tissue injury and articular cartilage degeneration of the OA joints and are associated with many pathological processes [10–12].

RAR*γ*, a member of retinoid receptors, belongs to the nuclear receptor (NRs) superfamily [13]. RAR*γ* forms as heterodimers with retinoid X receptors (RXRs) and then binding to the target retinoic acid response elements (RAREs) to regulate multiple genes expression in biological processes, such as cell growth, differentiation, and apoptosis of normal or malignant cells [14]. In addition to the classical genomic effects of RAR*γ*, it could also regulate gene expression through nongenomic effects in the absence or presence of ligands [15–17]. RAR*γ* also participates in cell inflammatory responses and plays an important role in acute and chronic inflammation [18].

Several studies have reported that RAR*γ* plays an essential role in cartilage matrix and proteoglycan homeostasis [19]. RAR*γ* activation could exhibit an antitumor effect on chondrosarcomas through promoting cartilage matrix degradation, inhibiting matrix synthesis, and inducing cell death [20, 21]. RAR*γ* could regulate the expression of specific genes in the differentiation of mice hypertrophic chondrocytes, such as Tg2, Mmp13, Col10A1, and Ccn2 [22]. All Trans Retinoic Acid (ATRA), the natural ligand for RARs, enables to increase the breakdown of cartilage and the nociceptive pain in OA, those effects can be blocked by the pan-RAR*γ* antagonist [23]. However, the clear and deep mechanisms of RAR*γ* in cartilage destruction of OA have not been investigated.

In this study, we aimed at investigating the effect of RAR*γ* on the destruction of cartilage in OA and explored the possible molecular mechanisms involved. The specific role of RAR*γ* in the inflammatory cytokines-induced cartilage damage process was uncovered in our study. Furthermore, we elucidate the function of RAR*γ* in the nosogenesis of OA and provide great value for the development of novel therapeutic strategies for the treatment of OA and related symptoms.

## 2. Materials and Methods

### 2.1. Materials and Reagents

The monosodium iodoacetate (MIA) (#I9148) and IKK inhibitor BMS-345541(#B9935) were obtained from Sigma-Aldrich (St. Louis, MO, USA). The recombinant human TNF-*α* (#300-01A) was obtained from PeproTech (Rocky Hill, NJ, USA). The sodium hyaluronate (SH) injection (#H20090719) was obtained from Seikagaku Corporation Takahagi Plant (Tokyo, Japan). The Retinoic Acid Receptor *γ* (RAR*γ*) agonist CD437 (#HY-100532) and antagonist LY2955303 (#HY-107765) were obtained from MedChemExpress (Monmouth Junction, NJ, USA). The primary antibodies against *α*-actinin (#11313-2-AP) and Flag (DYKDDDDK) tag (#20543-1-AP) were bought in Proteintech Group (Chicago, IL, USA). CCL4(#ab45690) and ADAMTS5(#ab41037) were obtained from Abcam (Cambridge, MA, USA). RAR*γ* (#8965S), STAT3 (#9139), p-STAT3(#9145), CERB (#9197), p-CREB (#9198), I*κ*B*α* (#4814), p-I*κ*B*α* (#2895), P65 (#8242), p-P65 (#3033), and MMP9 (#13667) antibodies were from Cell Signaling Technology (Danvers, MA, USA). All the secondary antibodies (goat antirabbit IgG (#AP132P) and goat antimouse IgG antibody (#AP124P)) used for western blots were obtained from Merck Millipore (Billerica, MA, USA). Fluorescein-conjugated secondary antibodies (Alexa Fluor 488 (#A21202) and Alexa Fluor 647 (#A31573)) were obtained from Invitrogen (Carlsbad, CA, USA).

### 2.2. Clinical Specimen

In the study, 7 normal human cartilage were extracted from the cartilage layer of the knee of seven patients with accidental amputation, including 4 males and 3 females, aged 23-44 years; while 20 OA cartilage tissues were provided by patients who underwent total knee replacement for OA, including 14 males and 6 females, aged 60-79 years. Patients diagnosed with OA in the study followed the international diagnostic criteria for OA established by the American College of Rheumatology Diagnostic Subcommittee. The following inclusion criteria were met for patients in this study: no history of NSAID or steroids at least 2 weeks prior to surgery; did not receive any relevant intra-articular injection for at least one month prior to surgery. All the patients were randomly selected to avoid selection bias. The study was approved by the Ethics Committee of the First Affiliated Hospital of Xiamen University in Fujian Province. All samples were collected with patient informed consent in accordance with the Hospital's code of ethics. All clinical research is carried out in full compliance with the principles of the Declaration of Helsinki.

### 2.3. Isolation and Culture of Primary Human Synovial Fibroblasts

In brief, synovial tissues of patients with accidental amputation and OA were dissected and washed 2-3 times with PBS. Then, the synovial tissues were minced into pieces of 2-3 mm^2^ and digested for 3 hours with 3 mg/mL collagenase II (#1148090, Sigma-Aldrich) in Hanks balanced salt solution at 37°C. Cell suspensions were filtered using a 70 *μ*m nylon filter. The synovial fibroblasts were cultured in medium supplemented with 1% antibiotic-antifungals and 10% fetal bovine serum (FBS). Passages 2 to 5 cells were used in all experiments.

### 2.4. Cell Culture and Treatment

The human chondrocyte C28/I2 was purchased from Sigma-Aldrich. HEK-293 and C28/I2 cells were cultured in DMEM (high glucose) supplemented with 10% FBS and 1% penicillin/streptomycin in a humidified atmosphere with 5% CO_2_ at 37°C. C28/I2 cells were stimulated with recombination human TNF-*α* (30 ng/mL) or IKK inhibitor BMS-345541 (10 *μ*M) for specific time, and then, the cells were collected for qRT-PCR and western blot analysis.

### 2.5. Plasmid Construction, Cell Transfection, and Lentivirus Production

RAR*γ* overexpression plasmid pLVX-Puro-RAR*γ* and pLVX-Puro-RAR*γ*-Flag, knockdown plasmid pLKO.1-Puro-RAR*γ* were bought from PPL (Public Protein/Plasmid Library, China). All the plasmids used in the experiment were confirmed by sequencing. For lentivirus packaging, HEK-293 cells were cotransfected with RAR*γ* or shRAR*γ* vector with packaging plasmid (psPAX2 and pMD2.G) by mixing with transfection reagent Lipofectamine 2000 (Invitrogen). After 48 hours of transfection, the virus was purified and collected, and then, the C28/I2 cells were infected.

### 2.6. Cell Viability Assay

The 5 × 10^3^ cells were plated on 96-well microplate for 24 hours. Then, the CCK8 solution (MedChemExpress) was added to the well and incubated in the incubator for 4 hours. Finally, the absorbance reader microplate (model 680, Bio-Rad, Hercules, CA, USA) was used to detect at the wavelength of 450 nm.

### 2.7. Immunofluorescence and Laser Confocal Microscopy

For immunofluorescence analysis, cells were plated on 35 mm confocal dishes to culture for 24 hours. Then, the cells were fixed with 4% paraformaldehyde for 15 min. Next, cells were permeabilized with Triton X-100 (0.1%) for 10 min and blocked with 5% goat serum for 1 hour. The cells were sequentially incubated with the primary antibody (rabbit anti-RAR*γ* (1 : 500), mouse anti-I*κ*B*α* (1 : 400)) at 4°C overnight, and fluorescein-conjugated secondary antibody (Alexa Fluor 488 (1 : 1000) and Alexa Fluor 647 (1 : 2000)) 1 hour darkly at room temperature. Finally, the cells were stained with DAPI and photographed by a confocal laser microscope (TCS SP8; Leica, Wetzlar, Germany).

### 2.8. Western Blotting

The cell total protein was lysed by RIPA buffer with protease inhibitor. Total protein was analyzed using 10 *μ*g, then separated by 10% SDS-PAGE gel and transferred to 0.22 *μ*m pore size PVDF membrane (Roche, Basel, Switzerland). After blocking the PVDF membrane with 5% nonfat milk, the primary antibody (rabbit anti-*α*-actinin (1 : 4000), rabbit anti-Flag (1 : 5000), rabbit anti-RAR*γ* (1 : 1000), mouse anti-STAT3 (1 : 1000), rabbit anti-p-STAT3 (1 : 1000), mouse anti-CERB (1 : 1000), rabbit anti-p-CREB (1 : 1000), mouse anti-I*κ*B*α* (1 : 1000), rabbit anti-p- I*κ*B*α* (1 : 1000), rabbit anti-P65 (1 : 1000), rabbit anti-p-P65 (1 : 1000), rabbit anti-CCL4 (1 : 1000), rabbit anti-ADAMTS5 (1 : 500), and rabbit anti-MMP9 (1 : 1000)) was incubated at 4°C overnight, and the HRP-conjugated secondary antibody was incubated at room temperature for 2 hours. Finally, the results were developed using chemiluminescence, examined in a ChemiDoc XRS+ system, and then analyzed using Image Lab™ software (Bio-RAD, Hercules, CA, USA).

### 2.9. Coimmunoprecipitation Assay (CO-IP)

Briefly, all cells were lysed by IP cell lysis buffer with protease inhibitor and centrifuged at 14,000 g for 10 minutes. Then, the cell lysates were incubated with primary antibodies (rabbit anti-RAR*γ* (1 : 50) and mouse anti-Flag (1 : 100) overnight at 4°C. Next, the magnetic beads coupled with protein A/G were added into the mixed solution and incubated at room temperature for 2 hours, and then, the magnetic beads were washed with IP wash buffer TBS four times and then eluted by 1× loading buffer to western blotting analysis.

### 2.10. Dual-Luciferase Reporter Assay

The RAR*γ* promoter region (relative to ATG initiation codon +2000 bp/-200 bp) was cloned into pGL3-Basic (Promega, Madison, WI, USA) to generate the luciferase reporter gene vector. In brief, HEK-293 cells were cotransfected with the corresponding reporter plasmid and internal control pRL-TK reporter constructs in each experiment. Dual-luciferase report assay (Promega) was applied for the determination of luciferase activities, which were normalized by renilla fluorescent activity to comparison.

### 2.11. Total RNA Extraction and Real-Time PCR Analysis

According to the manufacturer's instructions, the total cellular RNA was extracted using RNAsimple kit (TianGen, Beijing, China), and the RNA reverse transcription was prepared using the FastKing RT Kit (TianGen). qRT-PCR was performed on a 7500 Real-Time PCR System (ABI, Foster City, CA, USA) using SYBR reagent (TianGen) following the manufacturer's instructions. The relative expression data of each gene was calculated using 2^−ΔΔCT^ method, with ACTB as the internal reference gene. And the sequences of primer are shown in Table S1.

### 2.12. Immunohistochemistry (IHC), Hematoxylin and Eosin (H&E), and Safranin-O/Fast Green Staining

The tissue sections were dewaxed and rehydrated sequentially, followed by antigen repair and endogenous peroxidase inactivation. Donkey serum was then used to block nonspecific antigens in tissue sections. The primary antibody (rabbit anti-RAR*γ* (1 : 100)) was incubated overnight at 4°C, and the secondary antibody was incubated at room temperature for 2 h. Then, the sections were coloured with DAB, stained with hematoxylin again, dehydrated, and sealed. And for H&E staining and Safranin-O/fast green staining, the sections were subsequently stained by hematoxylin/eosin and safranin-o/fast green after deparaffinization and rehydration following the procedure. For the modified Mankin scale, two raters were arranged to score each sample independently according to the modified Mankin scale. The expression grading method of RAR*γ* evaluated by immunohistochemistry is described below. Two independent pathologists measured the IHC scores based on the percentages and intensity of the positive staining areas relative to the entire area or section. Staining scores were graded as follows: <10%, negative (-); 10–25%, weak (+); 26–50%, moderate (++); and >50%, strong (+++).

### 2.13. Animal Model and Treatment

All animals in this study were approved by the Animal Ethics Committee of Xiamen University and carried out in accordance with the animal experiment procedures formulated by Xiamen University Laboratory Animal Center. SPF grade SD rats, 6-8w, male, and weight 180-200 g were adapted to feeding for 7 days and randomly divided into 7 groups with 3 rats in each group. The rat model with degeneration of osteoarticular cartilage was established by injecting MIA into the articular cavity. Intra-articular injection of MIA is an effective method to induce OA in animals, it can be used to observe the early pathological changes of OA and study the effect of drugs on osteoarthropathy [24, 25]. The rats were randomly divided into 7 groups with 3 rats in each group: the healthy group which was only injected with physiological saline, MIA group, MIA plus RAR*γ* agonist CD437 (1 *μ*M) group, MIA with RAR*γ* antagonists LY2955303 (1 *μ*M), MIA with TNF-*α* (60 ng/mL) group, MIA with TNF-*α* and LY2955303 group, and MIA with sodium hyaluronate group which used for clinical OA treatment. 0.1 mL MIA solution (30 mg/mL) was injected into the joint cavity of rats, and normal saline was injected into the joint cavity of the control group. One week after injection, each group was given drug therapy, with 0.1 mL of drug solution injected into the joint cavity once a week for three weeks, during which the weight of the rats was strictly monitored. At the end of the experiment, the rats were sacrificed for cervical dislocation, removed whole blood to preserve serum, and measured the knee joint's diameter. The knee joint was removed and dissected to remove the surrounding excess tissue and leave the joint capsule intact. Then, the knee cartilage was fixed, EDTA decalcified, and paraffin embedded for subsequent experiments, or the cartilage layer of the fresh knee joint was stripped and tissue proteins were extracted for western blot analysis.

### 2.14. Quantification of Alkaline Phosphate (ALP) Concentration

Serum alkaline phosphate (ALP) concentration was measured by Alpl Assay Kit (#ab233466, Abcam) according to the manufacturer's instructions. The OD value was read at 405 nm and calculated and normalize according to the standard curve.

### 2.15. Online Database Analysis

The expression of RAR*γ* in normal cartilage tissue was analyzed by online database Pharos (https://pharos.nih.gov/) [26, 27]. To predict proteins interacting with RAR*γ*, the online database STRING (https://cn.string-db.org/) and GeneMANIA (http://genemania.org/) were used for analysis and mapping [28, 29].

### 2.16. Statistical Analysis

All values were expressed as mean ± SEM from at least three repeated independent experiments. The two-sided Student's *t*-test was used for comparison between two sets of normally distributed data. The statistical analysis of data was performed by GraphPad Prism (Version 9.3; La Jolla, CA, USA). *p* < 0.05 was considered to be statistically significant.

## 3. Results

### 3.1. RAR*γ* Was High Expression in Human OA Chondrocytes

**Figure 1:**
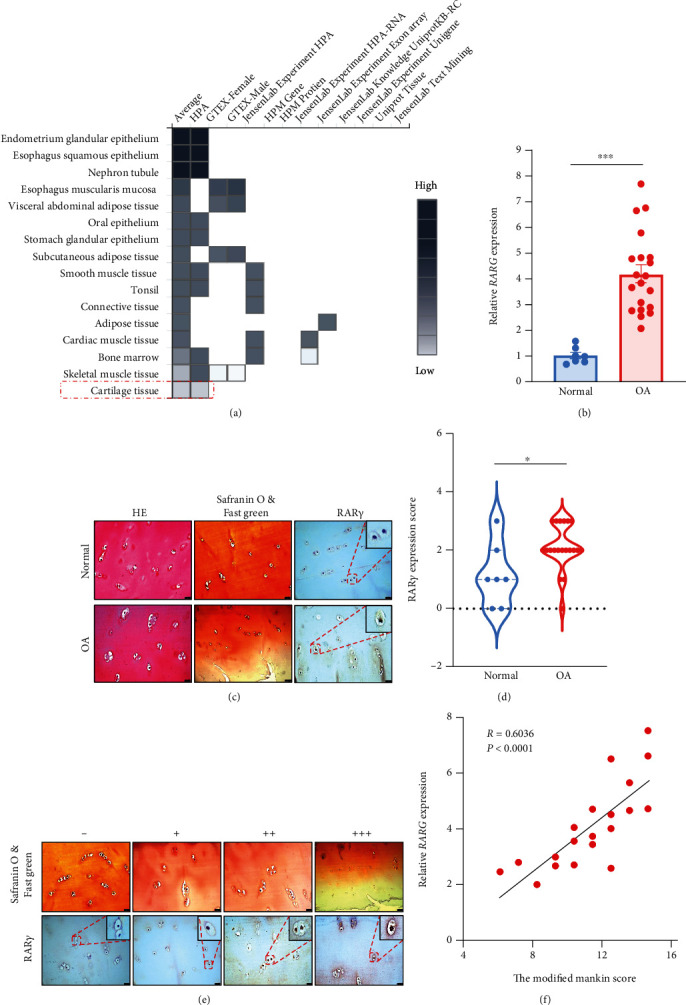
RAR*γ* was high expression in human OA chondrocytes compared with normal chondrocytes. (a) RAR*γ* expression in normal cartilage was analyzed by online database Pharos (https://pharos.nih.gov/).(b,c) Comparison of RAR*γ* expression in cartilage cells of healthy people (*N* = 7) and OA patients (*N* = 20) by qRT-PCR (b) and IHC (c). (d) Statistical and comparative analysis of RAR*γ* immunohistochemical staining scores in chondrocytes of healthy people and OA patients. (e) Relationship between the degree of cartilage damage and RAR*γ* expression grade in OA patients. (f) The RAR*γ* mRNA expression level was positively correlated with the Mankin scale of cartilage tissues (*N* = 20, *R* = 0.6036, *p* < 0.0001). Red dotted frames in ×400 show the corresponding zone magnification in ×1000. Data were mean ± SEM of three independent assays (^∗^*p* < 0.05; ^∗∗∗^*p* < 0.001).

In order to study the expression of RAR*γ* in human cartilage, bioinformatics analyses were carried out in database Pharos, and the results showed that RAR*γ* is shallow in normal cartilage (Figure 1(a)). RAR*γ* mRNA level was significantly increased in OA cartilage specimens compared with normal cartilage (Figure 1(b)). The IHC results also indicated that RAR*γ* was high expression in OA chondrocytes, and the localization of RAR*γ* in cartilage cells was both in nuclear and cytoplasm (Figure 1(c)). The expression of RAR*γ* in the cartilage of 8 normal people and 20 OA patients was analyzed and classified into four levels: negative (-), weak (+), medium (++), and strong (+++) expression. As shown in Table 1, the RAR*γ* expression in chondrocytes of OA patients was significantly higher than that of healthy people, and the IHC score of RAR*γ* also indicated the same results in Figure 1(d). In addition, RAR*γ* expression in OA patients was positively correlated with the degree of destruction (Figure 1(e)). And RAR*γ* mRNA expression in OA cartilage was positively correlated with the modified Mankin scale score (*R* = 0.6036, *p* < 0.0001) (Figure 1(f)). These findings suggested that RAR*γ* was high expression and had significant correlation in OA.

### 3.2. TNF-*α* Promoted the Expression of RAR*γ* through the NF-*κ*B Pathway

**Figure 2:**
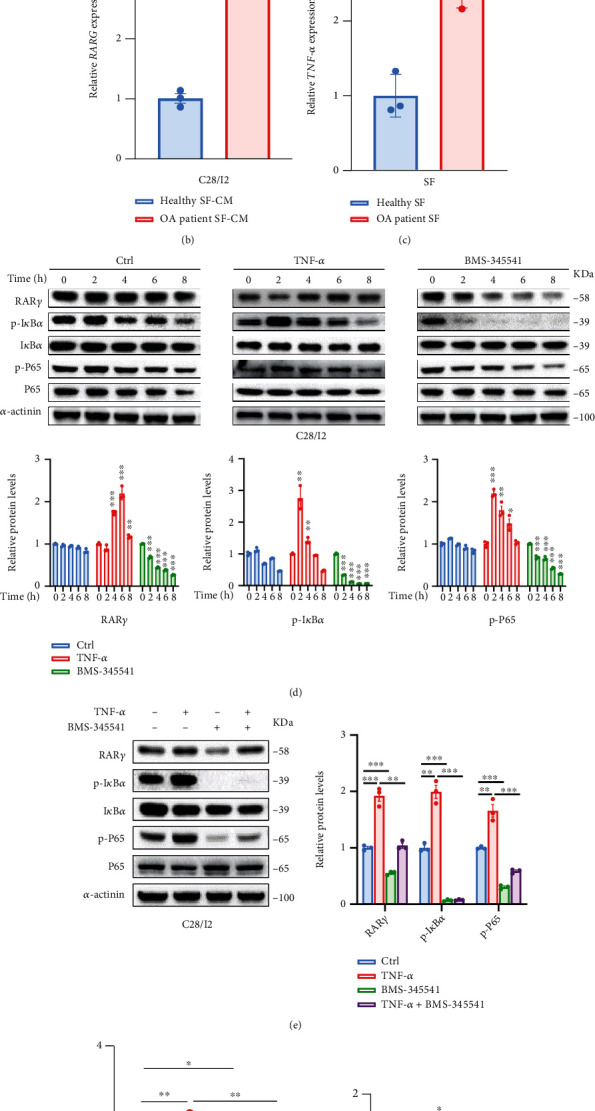
TNF-*α* promoted the expression of RAR*γ* by activating NF-*κ*B signaling pathway. (a,b) The 24 hours supernatant of synovial fibroblasts from healthy human and OA patients was cocultured with normal chondrocyte C28/I2 for 24 hours, respectively. Then, the activation of NF-*κ*B signaling pathway and the expression of RAR*γ* in C28/I2 cells were detected by western blot (a) and qRT-PCR (b). (c) The expression of TNF-*α* in synovial fibroblasts of healthy persons and OA patients was detected by qRT-PCR. (d) After C28/I2 cells were treated with TNF-*α* (30 ng/mL) or IKK inhibitor BMS-345541 (10 *μ*m) at different time points, the expression of RAR*γ* and NF-*κ*B signaling pathway were examined by western blot. (e,f) After the C28/I2 cells were treated with TNF-*α* (30 ng/mL) and BMS-345541 (10 *μ*m) separately or in combination, changes in NF-*κ*B signaling pathway and the expression of RAR*γ* in C28/I2 cells were detected by western blot and qRT-PCR. (g) HEK-293 cells were transfected with RAR*γ* promoter reporter plasmid and pRL-TK reporter internal reference plasmid; the fluorescence intensity of renilla (RL) and firefly (FL) was detected after 4 hours of treatment with TNF-*α* (30 ng/mL) and BMS-345541 (10 *μ*m), respectively, or in combination, and the values were normalized. Data were mean ± SEM of three independent assays (^∗^*p* < 0.05; ^∗∗^*p* < 0.01; and ^∗∗∗^*p* < 0.001).

To further explored the mechanism of RAR*γ* overexpression in OA chondrocytes, the synovial fibroblasts, extracted from knee synovial tissues of normal and OA patients during knee surgery, were used to coculture with normal chondrocyte C28/I2 for 48 hours. The results indicated that the mRNA level of RAR*γ* was elevated both in gene and protein in C28/I2 cells which were treated with the supernatant culture medium of OA synovial fibroblasts (Figures 2(a) and 2(b)). And the level of p-I*κ*B*α* and p-P65 were elevated in the C28/I2 cell. Next, the mRNA level inflammatory cytokines TNF-*α* were elevated in OA synovial fibroblasts (Figure 2(c)).

In addition, the expression of RAR*γ* in C28/I2 cells was significantly increased after being treated with recombination human TNF-*α* protein along with the activation of NF-*κ*B pathway (Figure 2(d)). On the contrary, when the inhibitor of NF-*κ*B pathway BMS-345541 (a highly selective inhibitor of the catalytic subunits of IKK-2 and IKK-1) was used to treat C28/I2 cell, the protein level of RAR*γ* and the activation of NF-*κ*B pathway was downregulated in a time-dependent manner (Figure 2(d)). Moreover, when C28/I2 cells were treated with a combination of TNF-*α* and BMS-345541, the expression of RAR*γ* could be rescued compared with the BMS-345541 group and decreased compared with TNF-*α* group (Figures 2(e) and 2(f)). The activation of the NF-*κ*B pathway whether could increase the transcription of RAR*γ* genes was evaluated by dual-luciferase reporter assay. As shown in Figure 2(g), the RAR*γ* promoter luciferase activity was obviously increased in TNF-*α* group and inhibited in BMS-345541 group, the combination of TNF-*α* and BMS-345541 put it in the middle. Here, our data indicated that TNF-*α* could activate the NF-*κ*B pathway to promote the transcription of RAR*γ*.

### 3.3. The Damage of Cartilage Matrix Was Induced by Overexpression of RAR*γ*

**Figure 3:**
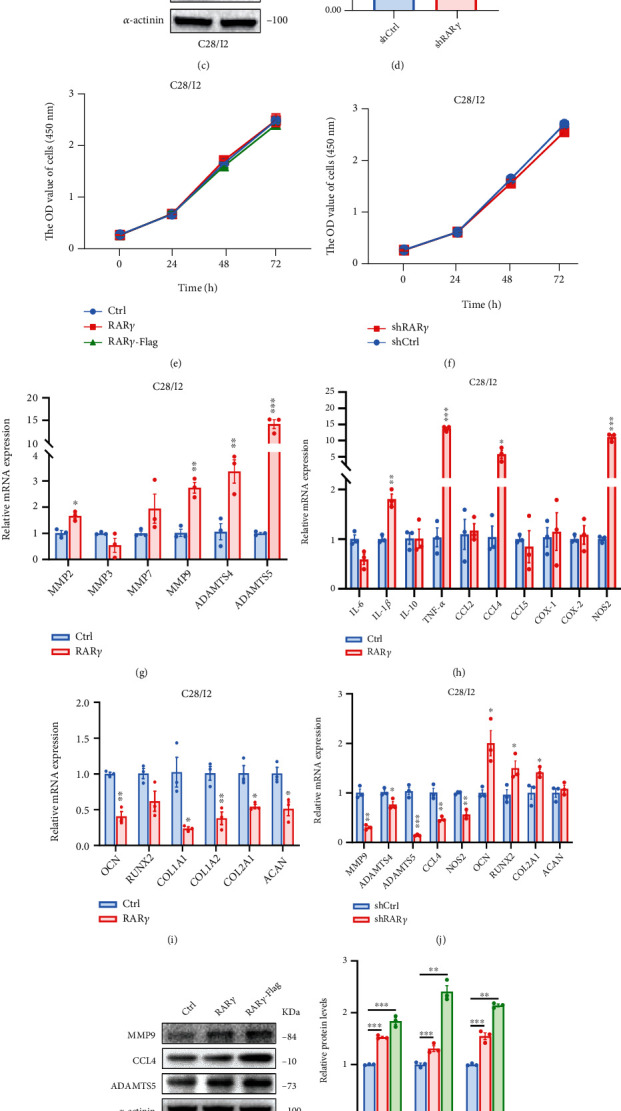
RAR*γ* induced the damage of cartilage matrix in cartilage cells. (a–d) RAR*γ* overexpression (a,b) and knockdown (c,d) C28/I2 cell lines were constructed and verified by western blot and qRT-PCR. (e,f) CCK8 assay was used to detect the effects of RAR*γ* overexpression (e) and knockdown (f) on proliferation of C28/I2 cells. (g–j) qRT-PCR was used to detect the changes of ECM degradation (g), inflammation (h), differentiation, and collagen (i) related genes after RAR*γ* overexpression in C28/I2 cells. (j) The effects of knockdown RAR*γ* on related gene levels in C28/I2 cell lines were detected by qRT-PCR. (k,l) RAR*γ* overexpression and knockdown protein expression of MMP9, CCL4, and ADAMTS5 were analyzed by western blot. Data were mean ± SEM of three independent assays (^∗^*p* < 0.05; ^∗∗^*p* < 0.01; and ^∗∗∗^*p* < 0.001).

The effect of the expression disorder of RAR*γ* in OA cartilage cells is still unknown. RAR*γ* overexpression (RAR*γ* and RAR*γ*-flag) (Figures 3(a) and 3(b)) or knockdown (Figures 3(c) and 3(d)) C28/I2 cell model were established and verified to explore the exact role of RAR*γ* in OA. Cell viability was assessed by CCK8, which indicated no growth difference when overexpression or knockdown RAR*γ* (Figures 3(e) and 3(f)). And the damage of cartilage matrix is the main feature of OA; we detected the expression of genes related to the degradation of cartilage matrix, and the result showed that the overexpression of RAR*γ* could increase the expression of MMP2, MMP7, MMP9, ADAMTS4, or ADAMTS5 (Figure 3(g)). Inflammatory cytokine and chemokines related genes like IL-1*β*, TNF-*α*, CCL4, and NOS2 were significantly increased in RAR*γ* overexpression group (Figure 3(h)). But the genes about differentiation OCN, generation collagen, and aggrecan COL1A1, COL1A2, COL2A1, and ACAN of cartilage cells were downregulated when overexpression RAR*γ* (Figure 3(i)). In the next, we validated some genes which were significantly changed in RAR*γ* overexpression cells. On the contrary, the expression of MMP9, ADAMTS4, ADAMTS5, CCL4, and NOS2 was decreased and OCN, COL2A1, ACAN were increased after knockdown of RAR*γ* in C28/I2 cells (Figure 3(j)). And the protein expression of MMP9, ADAMTS5, and CCL4 was also increased in RAR*γ* overexpression group (Figure 3(k)) and was opposite in RAR*γ* knockdown group (Figure 3(l)). The results above indicated that a high expression level of RAR*γ* can promote the expression of genes about degradation of matrix, inflammation, and inhibit expression of genes related to differentiation, secretion of collagen, and aggrecan.

### 3.4. RAR*γ* Promoted the Degradation of Cartilage Cells through the Activation of NF-*κ*B Pathway

**Figure 4:**
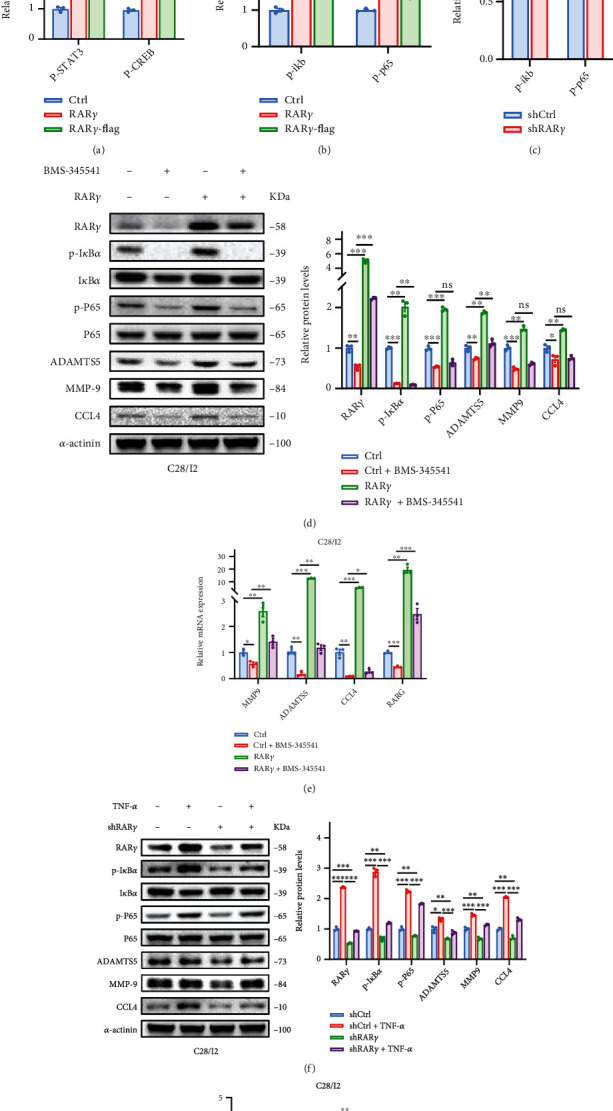
RAR*γ* induces chondrocytes degradation by activating NF-*κ*B signaling pathway. (a–c) Effects of RAR*γ* overexpression (a,b) and knockdown (c) on related signaling pathways in C28/I2 cells. (d,e) RAR*γ* was overexpressed in C28/I2 and treated with BMS-345541 (10 *μ*m) for 4 hours. The activation of NF-*κ*B signaling pathway and the expression of related genes were detected by qRT-PCR and western blot. (f,g) RAR*γ* was knockdown in C28/I2 and treated with TNF-*α* (30 ng/mL) for 4 hours. Activation of NF-*κ*B signaling pathway and expression of related genes were detected by qRT-PCR and western blot. Data were means ± SEM of three independent assays (^∗^*p* < 0.05; ^∗∗^*p* < 0.01; and ^∗∗∗^*p* < 0.001).

The alternation of signaling pathways was further studied in RAR*γ* overexpression chondrocytes. The results indicated that the overexpression of RAR*γ* could increase the level of p-STAT3, p-CREB, p-I*κ*B*α*, and p-P65 (Figures 4(a) and 4(b)). There are many studies that demonstrated that NF-*κ*B pathway is an important signaling pathway that controls normal development and the pathological destruction of cartilage [30–32]. Hence, we speculated that RAR*γ* exserts its functions in chondrocytes by regulating the activity of NF-*κ*B pathway. Knockdown of RAR*γ* also impeded the activation of NF-*κ*B pathway (Figure 4(c)). Furthermore, the mechanism was further demonstrated by adding NF-*κ*B pathway inhibitor BMS-345541 in RAR*γ* overexpression cells and activator TNF-*α* in RAR*γ* knockdown cells. BMS-345541 blocked the NF-*κ*B pathway and abolished the upregulation of targets genes MMP9, ADAMTS5, and CCL4 which was brought by RAR*γ* overexpression (Figures 4(d) and 4(e)). In addition, TNF-*α* activated the NF-*κ*B pathway and rescued the downregulation of targets genes above which was brought by RAR*γ* knockdown (Figures 4(f) and 4(g)). Therefore, the above results indicated that expression of RAR*γ* would affect the activation of NF-*κ*B pathway to regulate the downstream genes about OA progression.

### 3.5. RAR*γ* Activated the NF-*κ*B Signaling Pathway by Interacting with I*κ*B*α* in Cartilage Cells

**Figure 5:**
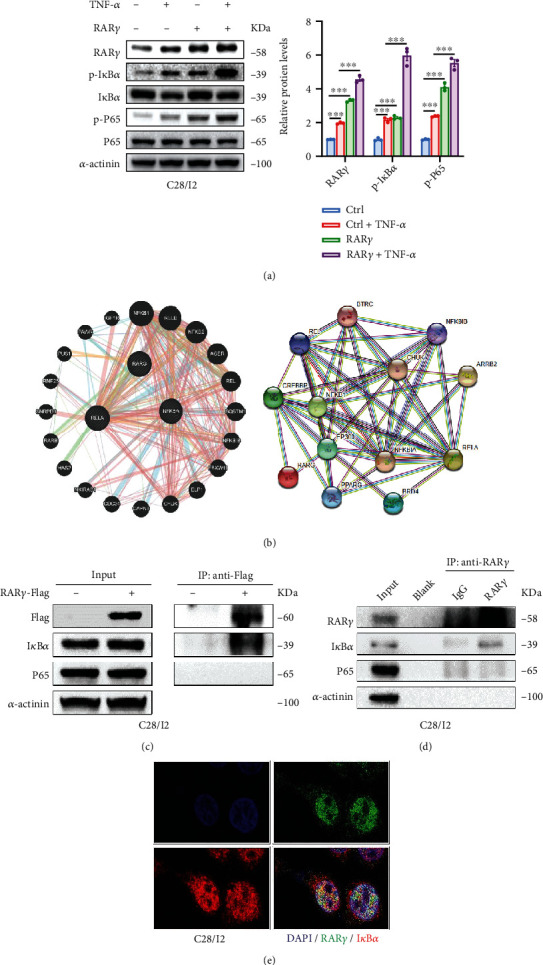
RAR*γ* interacted with I*κ*B*α* and promoted the activation of NF-*κ*B signaling pathway. (a) C28/I2 cells overexpressing RAR*γ* were treated with TNF-*α* (30 ng/mL) for 4 hours, and the activation of NF-*κ*B signaling pathway was detected by western blot. (b) Online public databases predict that RAR*γ* may interact with I*κ*B*α* and P65 proteins in NF-*κ*B signaling pathways (STRING, https://cn.string-db.org/and GeneMANIA, http://genemania.org/). (c) CO-IP assay was used to verify whether the exogenous overexpressed RAR*γ*-Flag protein interacts with I*κ*B*α* or P65. (d) The CO-IP assay was further used to verify whether the endogenous RAR*γ* protein interacted with I*κ*B*α* or P65. (e) The colocalization of RAR*γ* and I*κ*B*α* in C28/I2 cells was detected by immunofluorescence. Magnification in ×1000.

We have demonstrated that the abnormally high expression of RAR*γ* promoted the activation of NF-*κ*B pathway. And this activation effect was enhanced with the addition of TNF-*α*, which represented a higher level of p-I*κ*B*α* and p-P65 (Figure 5(a)). We hypothesized whether RAR*γ* could increase the activation of NF-*κ*B pathway through interacting with I*κ*B*α* or P65, which had been predicted by online protein interaction databases (Figure 5(b)). The CO-IP assay of RAR*γ*-Flag C28/I2 cells was performed to verify this speculation, and the result indicated that RAR*γ*-flag could interact with I*κ*B*α* but not P65 (Figure 5(c)). The endogenous CO-IP assay which IP by RAR*γ* antibody also got the same result (Figure 5(d)). Furthermore, the RAR*γ* and I*κ*B*α* were colocated to the cytoplasm and nuclear in the C28/I2 cells verified by immunofluorescence (Figure 5(e)). Taken together, the results demonstrated that RAR*γ* could promote the activation of NF-*κ*B pathway through interacting with I*κ*B*α* and form a positive feedback loop in chondrocyte C28/I2.

### 3.6. RAR*γ* Affected the Progression of OA in SD Rat Model

**Figure 6:**
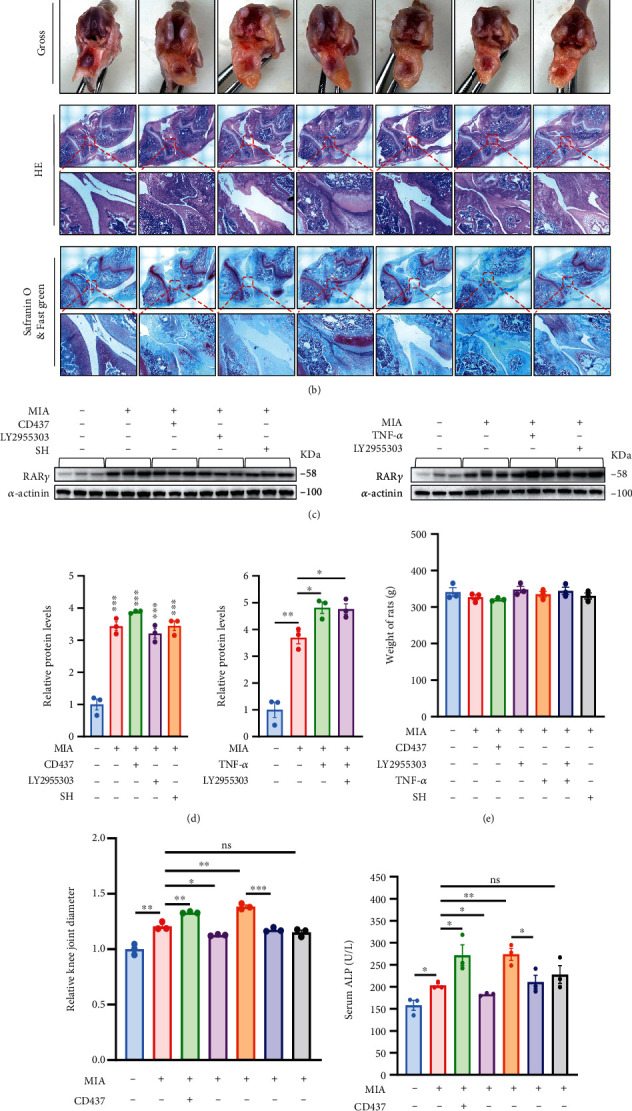
*In vivo* study of the effects of RAR*γ* in the progression of OA in SD rat model. (a) Establishment of OA model and subsequent drug treatment in rats. Randomly divided into 7 groups with 3 rats in each group. 0.1 mL monosodium iodoacetate solution (30 mg/mL) or normal saline was injected into the joint cavity of rats. One week after injection, 0.1 mL of the indicated drug was injected into the joint cavity once a week for three weeks. The drug including CD437 (1 *μ*m), LY2955303 (1 *μ*m), TNF-*α* (60 ng/mL), and SH. (b) Representative rat joints were shown, a gross view (upper) of the knee joint after dissection, and HE (middle) and safranin-o/fast green (down) staining were used for pathological analysis. (c,d) The expression of RAR*γ* in each group was analyzed by western blot. (e) Body weight statistics of rats in each group. (f) Knee joint swelling degree, measure knee joint diameter. (g) Serum alkaline phosphatase activity was measured. Data were mean ± SEM of three independent assays. Magnification in ×50. (^∗^*p* < 0.05; ^∗∗^*p* < 0.01; and ^∗∗∗^*p* < 0.001).

The rat model with degeneration of osteoarticular cartilage was established to confirm the therapeutic effect of RAR*γ* in OA (Figure 6(a)). To observe the early pathological changes of OA and study the effect of drugs on osteoarthropathy, the MIA-induced rat model was selected for experiment. Rats were randomly divided into 7 groups which was mentioned in Methods. The establishment of the OA rat model and the drug treatment process were shown in Figure 6(a).

The knees were dissected and representative photographs were taken after sacrificing of rats (Figure 6(b) upper). The results of hematoxylin/eosin and safranin-o/fast green staining were shown in Figure 6(b). Compared with healthy control, the MIA had severe joint damage, especially in femoral condylar cartilage layer. In clinical manifestations of MIA-induced OA, rats treated with RAR*γ* agonist CD437 showed significant destruction, including significant destruction of cartilage degradation, significant increase in proteoglycan loss, and reduction in the number of chondrocytes, while mice treated with RAR*γ* antagonist LY2955303 showed relatively substantial cartilage protection. Besides, the inflammatory cytokine TNF-*α* could aggravate the destruction of cartilage, same as CD437, and LY2955303 also could rescue the damage of TNF-*α*. Sodium hyaluronate as a joint lubricant reduces the symptoms but not the damage. And compared with the healthy group, the expression of RAR*γ* in the joint articular cartilage of rat models was significantly increased in MIA group, as well as in the CD437, LY2955303, and sodium hyaluronate (SH) groups. Moreover, the expression of RAR*γ* increased more significantly in the TNF-*α* group than in the MIA group (Figures 6(c) and 6(d)). The body weight of rats had no significant difference between the groups (Figure 6(e)). The diameter of the knee joint was also measured and the degree of swelling was also consistent with the damage of cartilage layer (Figure 6(f)). Besides, the serum concentrations of alkaline phosphatase (ALP) as an indicator that could indirectly reflect destruction of cartilage were quantified (Figure 6(g)). The serum ALP level of rats was also consistent with the destruction of cartilage layer in OA.

In summary, RAR*γ* was high expression in OA rat cartilage and activation of it could promote the progression of OA, but the inhibition of RAR*γ* could rescue the damage brought by TNF-*α*. Our data provided effective clinical treatment strategies for OA in future research.

## 4. Discussion

**Figure 7:**
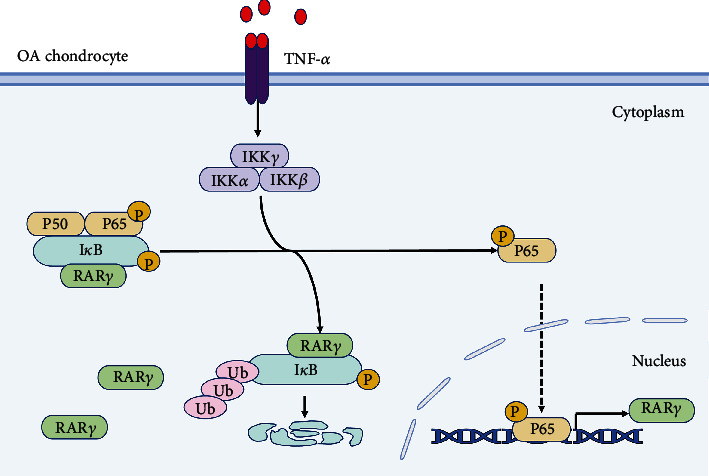
(a) Illustrate the mechanism of the study. When OA occurs, synovial fibroblasts secrete the validation factor TNF-*α* into the articular cavity, which promotes the activation of TNF-*α* signaling pathway in chondrocytes and leads to an increased expression of RAR*γ* downstream. The increased expression of RAR*γ* in chondrocytes in turn assists the activation of NF-*κ*B signaling pathway, thus forming a positive feedback loop that leads to downstream gene expression related to cartilage matrix degradation and inflammation, thus aggravating the development of OA.

OA, as the most common degenerative joint disease, could cause joint pain and loss of function, posing a major threat to the health of the elderly [33]. Although, lots of effective treatment methods for OA have been developed, including drug therapy and surgery, there is still a lack of more effective methods to bring long-term benefits to patients [34, 35]. Therefore, exploring the pathogenesis of OA is of great significance for the prevention and treatment of OA. Our study revealed that the RAR*γ* was significant elevation in OA patients' cartilage tissues and TNF-*α*-induced human chondrocytes. And RAR*γ* overexpression promoted the matrix degradation and inflammatory response in chondrocytes. More importantly, we demonstrated that the NF-*κ*B pathway plays an irreplaceable role in the action of RAR*γ*-caused dysfunction of chondrocytes and RAR*γ* interacts with I*κ*B*α* to form a positive feedback loop to aggravate cartilage destruction (Figure 7(a)).

The pathogenesis and mechanism of OA are extremely complicated, mainly involving mechanical, inflammatory, and metabolic factors, which ultimately lead to the structural destruction and death of joint cartilage and synovium [36]. RAR*γ* as a nuclear receptor has been confirmed to be involved in many critical biological processes [37]. However, the role and mechanism of RAR*γ* in OA are still unknown. Here, we demonstrated that RAR*γ* was significantly high expression in the cartilage cells of OA patients and increased with the severity of OA. Those results laid a foundation for the follow-up study on the role of RAR*γ* in OA.

After cartilage injury, the proinflammatory mediators and products are synthesized and elevated in chondrocytes. They would stimulate hyperplasia and proinflammatory responses of adjacent synovium, and the synovial fibroblasts also release proinflammatory products which lead to the dysfunction of chondrocytes [38]. Proinflammatory cytokines disrupt cartilage homeostasis mainly by promoting the catabolism activity and blocking the anabolism activity of chondrocytes, inhibiting the synthesis of collagen and proteoglycan and increasing the expression of MMPs and ADAMTSs [39, 40]. In our study, OA synovial fibroblasts highly expressed proinflammatory TNF-*α* and the activation of the NF-*κ*B pathway results in high expression of RAR*γ* in chondrocytes. We also found that RAR*γ* was a target gene of NF-*κ*B pathway. This explained that the overexpression of RAR*γ* in OA was caused by the activation of inflammatory signaling pathway in chondrocytes, which was involved in the regulation of cartilage homeostasis.

One of the main features of OA is cartilage degeneration, which manifests as the degradation of collagen in cartilage tissue [41]. MMPs and ADAMTSs play a crucial role in the matrix degradation of OA, and the increased expression of these enzymes can directly participate in and aggravate the pathogenesis of OA [42, 43]. We found that the RAR*γ* overexpression increased the expression of MMPs (MMP2, MMP7, and MMP9) and ADAMTSs (ADAMTS5 and ADAMTS4). And the expression of inflammatory chemotaxis and stress-related genes (IL-1*β*, TNF-*α*, CCL4, and NOS2) in chondrocytes was also increased after RAR*γ* overexpression. For collagen, aggrecan generation, and differentiation OCN, RUNX2, COL1A1, COL1A2, COL2A1, and ACAN were all decreased after RAR*γ* overexpression. The expression of RAR*γ* had no effect on the growth of chondrocytes, but RAR*γ* could increase the degradation of the extracellular matrix (ECM) and promote inflammation and stress of chondrocytes. RAR*γ* also inhibited chondrocyte differentiation and collagen glycoprotein synthesis to accelerate cartilage destruction further.

It has been reported that RAR*γ* could exert nongenomic effects to regulate various phenotypes of cells by activation and inactivation of various signaling pathways [44–46]. RAR*γ* also is an excellent partner to interact with other protein which is the crucial regulator of some important signaling pathways. And it has also been reported that cytoplasmic RAR*γ* can interact with RIP1 to form the death complex IIa and participate in the cell death process induced by RIP1, which reveals a key checkpoint for the cell survival and death signal conversion mechanism triggered by RIP1. [47]. We found that IL-6/STAT3, CREB, or NF-*κ*B pathway could be activated by RAR*γ* in chondrocytes. NF-*κ*B signaling pathway could be activated by the proinflammatory cytokines, mechanical stress, and extracellular matrix degradation products and which results in affecting cartilage matrix remodelling, chondrocyte apoptosis, and synovial inflammation [48, 49]. Targeting the NF-*κ*B signaling pathway has provided new potential therapeutic strategies for the treatment of OA. And the further study also demonstrated NF-*κ*B pathway was an essential pathway for RAR*γ* to regulate downstream genes like MMP9, ADAMTS5, and CCL4. Hence, RAR*γ* increased the activation of NF-*κ*B pathway contributing to aggravating cartilage damage and inflammation.

Furthermore, RAR*γ* collaborated with TNF-*α* to activate the NF-*κ*B signaling by interacting with I*κ*B*α* instead of P65 and exerts regulatory role functions through nongenomic effects. We speculated that RAR*γ*, interacting with I*κ*B*α*, could suppress the ubiquitination degradation of p-I*κ*B*α* to promote the activation of NF-*κ*B pathway. TNF-*α* promoted the expression of RAR*γ* through NF-*κ*B pathway, and RAR*γ* with high expression promoted the TNF-*α* expression and interact with I*κ*B*α* to further promote the activation of NF-*κ*B pathway, making RAR*γ* and NF-*κ*B pathway form a positive feedback loop in chondrocytes. This positive feedback loop leads to the worsening of OA under inflammatory conditions.

*In vivo* model of OA, the treatment of RAR*γ*-specific agonists and TNF-*α* worsened OA, and the treatment of RAR*γ*-specific inhibitors slowed joint damage associated with both. Besides, the expression of RAR*γ* was increased in OA and TNF-*α* groups. Animal models also reflect the existence of positive feedback loops formed by RAR*γ* and NF-*κ*B pathway, which could promote the severity of OA. These results suggested that the cartilage destruction and inflammatory response of OA patients were perhaps greatly alleviated by blocking of RAR*γ*.

## 5. Conclusions

In conclusion, the present study revealed that the joint inflammation in OA patients could promote the expression of RAR*γ* in chondrocytes. Highly expressed RAR*γ* can further assist in increasing the activation of NF-*κ*B pathways caused by inflammation, thus forming a positive feedback loop composed of RAR*γ* and NF-*κ*B pathways. Activation of this loop can lead to the progression of OA, such as degradation of cartilage matrix and increased inflammatory response of chondrocytes. However, there are still some results worthy of further studies, such as how RAR*γ* and I*κ*B*α* interact to affect I*κ*B*α* phosphorylation and how to regulate downstream-related proteins. Taken together, our results reveal that RAR*γ* proteins may be promising as a potential therapeutic target for OA and provide a new research strategy for the targeted treatment of OA.

## Figures and Tables

**Table 1:** The expression of RAR*γ* in human normal and OA cartilage tissue (^∗∗^*p* < 0.01).

	Total	The expression of RAR*γ*		*X* ^2^	*p*
		Low (−, +)	High (++, +++)		
Normal	7	5	2	7.9191	0.0049^∗∗^
OA	20	3	17		

## Data Availability

The data used to support the findings of this study are available from the corresponding author upon request.

## Supplementary Materials

Supplementary Materials The primer sequences were presented in Table S1.
