# Supplementary material for: Retinoic Acid Receptor Gamma (RARγ) Promotes Cartilage Destruction through Positive Feedback Activation of NF-κB Pathway in Human Osteoarthritis
Source: Mediators Inflamm. 2022 Nov 7;2022:1875736. doi: 10.1155/2022/1875736 (PMC9663240; doi:10.1155/2022/1875736)
Supplement: Supplementary Materials — The primer sequences were presented in Table S1. [file 1875736.f1.docx]

**Table S1.** the primer sequence of genes.

| **Gene Primers** | **Sequence 5’-3’** |
| --- | --- |
| ***RARG-F*** | CAGCACTAAGGGAGCTGAAA |
| ***RARG-R*** | CTCCAGCATCTCTCGGATTAAG |
| ***MMP2-F*** | CCCACTGCGGTTTTCTCGAAT |
| ***MMP2-R*** | CAAAGGGGTATCCATCGCCAT |
| ***MMP3-F*** | CAGGCTTTCCCAAGCAAATAG |
| ***MMP3-R*** | CTCCAACTGTGAAGATCCAGTAA |
| ***MMP7-F*** | GAGTGAGCTACAGTGGGAACA |
| ***MMP7-R*** | CTATGACGCGGGAGTTTAACAT |
| ***MMP9-F*** | AGACCTGGGCAGATTCCAAAC |
| ***MMP9-R*** | CGGCAAGTCTTCCGAGTAGT |
| ***ADAMTS4-F*** | CGCTTTGCTTCACTGAGTAGAT |
| ***ADAMTS4-R*** | CTGTTAGCAGGTAGCGCTTTAG |
| ***ADAMTS5-F*** | ACTACGATGCAGCTATCCTGT |
| ***ADAMTS5-R*** | GTCCCAACGTCTGCCATTC |
| ***IL-6-F*** | CCTGAACCTTCCAAAGATGGC |
| ***IL-6-R*** | TTCACCAGGCAAGTCTCCTCA |
| ***IL-1β-F*** | ATGATGGCTTATTACAGTGGCAA |
| ***IL-1β-R*** | GTCGGAGATTCGTAGCTGGA |
| ***IL-10-F*** | GCTGGAGGACTTTAAGGGTTAC |
| ***IL10-R*** | GATGTCTGGGTCTTGGTTCTC |
| ***TNF-α-F*** | GAGGCCAAGCCCTGGTATG |
| ***TNF-α-R*** | CGGGCCGATTGATCTCAGC |
| ***CCL4-F*** | CCAGCCAGCTGTGGTATT |
| ***CCL4-R*** | CAGTTCAGTTCCAGGTCATACA |
| ***CCL5-F*** | TGCCCACATCAAGGAGTATTT |
| ***CCL5-R*** | GATGTACTCCCGAACCCATTT |
| ***COX-1-F*** | CACTTCACCCACCAGTTCTT |
| ***COX-1-R*** | CGCTCCAGATTGTCTCCATAAA |
| ***COX-2-F*** | CTCAGCCATACAGCAAATCCT |
| ***COX-2-R*** | CCGGGTACAATCGCACTTAT |
| ***NOS2-F*** | GTCAGAGTCACCATCCTCTTTG |
| ***NOS2-R*** | GCAGCTCAGCCTGTACTTATC |
| ***OCN-F*** | CAGGCGCTACCTGTATCAAT |
| ***OCN-R*** | CGATGTGGTCAGCCAACT |
| ***RUNX2-F*** | TGGTTACTGTCATGGCGGGTA |
| ***RUNX2-R*** | TCTCAGATCGTTGAACCTTGCTA |
| ***COL1A1-F*** | CGATGGATTCCAGTTCGAGTATG |
| ***COL1A1-R*** | CTTGCAGTGGTAGGTGATGTT |
| ***COL1A2-F*** | AGAGTGGAGCAGTGGTTACTA |
| ***COL1A2-R*** | GATACAGGTTTCGCCAGTAGAG |
| ***COL2A1-F*** | CAGGATGGGCAGAGGTATAATG |
| ***COL2A1-R*** | GAGGCAGTCTTTCACGTCTT |
| ***ACAN-F*** | AACGGTCTACCTCTACCCTAAC |
| ***ACAN-R*** | GGAGAAGGAACCGCTGAAAT |
| ***ACTB F*** | GGACCTGACTGACTACCTCAT |
| ***ACTB R*** | CGTAGCACAGCTTCTCCTTAAT |
